# Editorial: Management of Immune-Related Adverse Events for Patients Undergoing Treatment With Checkpoint Inhibitors

**DOI:** 10.3389/fonc.2019.00365

**Published:** 2019-05-08

**Authors:** Bernardo Leon Rapoport

**Affiliations:** ^1^Department of Immunology, Faculty of Health Sciences, University of Pretoria, Pretoria, South Africa; ^2^The Medical Oncology Centre of Rosebank, Johannesburg, South Africa

**Keywords:** immune related adverse effects, colitis, pneumonitis, anti CTLA 4, anti-PD 1

Immunotherapy with immune checkpoint inhibitors has emerged as the most significant advance in the treatment of cancer in recent years and has revolutionized cancer management (1). Until recently, it had been assumed that the immune system was not effective in protecting humans against the development of neoplastic diseases. Checkpoints inhibitors are co-receptors expressed by T cells. These co-receptors regulate T cell activation negatively and play a central role in the maintenance of peripheral self-tolerance. Co-inhibitory receptor ligands are significantly expressed in a variety of malignancies resulting in evasion of anti-cancer immunity. These molecules include programmed cell death protein 1 (PD-1) and cytotoxic T lymphocyte-associated antigen 4 (CTLA-4) and were discovered by Tasuku Honjo and James P. Allison in 1992 and 1996, respectively (2, 3). These scientists were jointly awarded the 2018 Nobel Prize for Physiology or Medicine in recognition of this ground-breaking research. Monoclonal antibodies targeting the CTLA-4 and PD-1 and their ligands have produced significant clinical responses against a variety of malignancies (4). FDA registered checkpoint inhibitors include pembrolizumab (5), nivolumab (6), cemiplimab (7), atezolizumab (8), darvolumab (9) and avelumab (10) for numerous indications including melanoma, lung cancer (small and non-small cell types), bladder cancer, Hodgkin's disease and others (5–10). Other co-inhibitory molecules under research include T cell immunoglobulin and mucin domain-containing molecule-3 (TIM-3) (11), Lymphocyte activation gene-3 (LAG-3) (12), V-domain Ig-containing Suppressor of T cell Activation (VISTA) (13), and B- and T-lymphocyte attenuator (BTLA) (14). Treatment with antibodies inhi biting immune checkpoints are well-tolerated by the vast majority of patients and are less toxic compared to standard anticancer chemotherapy agents. These immune side-effects are referred to as immune-related adverse events (IrAE) (15).

These toxicities include fatigue, dermatological, gastrointestinal, hepatic, pulmonary, endocrine, ocular, neurological, and rare toxicities such as diabetes, cardiac and hematological. Dermatological toxicities can appear following the first dose of an immune checkpoint inhibitor and can be ongoing. These rashes are frequently maculopapular and mild in nature (16). Rash, and generalized pruritus occur more commonly with CTLA-4 inhibitors compared to anti-PD-1 inhibitors (17). Rare cases of serious skin reactions such as Stevens-Johnson syndrome and toxic epidermal necrolysis have been reported (18). The development of vitiligo occurs in a small percentage of patients receiving immunotherapy with checkpoint inhibitors and is associated with long term survival and clinical benefit (19).

Gastrointestinal side effects can occur in the form of mucositis, aphthous ulcers, gastritis, colitis, and abdominal pain. Diarrhea, with blood or mucus in the stool, can be observed. In severe cases, these complications can evolve to toxic megacolon and perforation and must be ruled out in patients with peritonitis symptoms (20). Other infectious causes of diarrhea such as Clostridium difficile infection can be associated in severe cases (20).

Immune-related pneumonitis is a serious IrAE reported in patients undergoing immune checkpoint inhibition. Pneumonitis is more common with PD-1 and PDL-1 blockers, however the incidence is < 1% and presents later during the treatment phase (21). Patients undergoing immunotherapy, experiencing new symptoms of dyspnea or cough, should alert the clinician. This complication could be fatal (21).

Endocrine IrAE symptoms are generally non-specific and include fatigue, mental state changes, headaches and dizziness related to hypotension (22). Hypophysitis and hypothyroidism are the most common abnormalities documented (22). Clinicians should screen for thyroid abnormalities and baseline thyroid function tests. Other hormone assays may be indicated in some patients. Ophthalmological IrAE in the form of mild, moderate or severe episcleritis, uveitis or conjunctivitis has been described (23). Neurological IrAE includes posterior reversible encephalopathy syndrome, aseptic meningitis, enteric neuropathy, transverse myelitis, and Guillain-Barre syndrome (24).

Less frequent IrAE's include red cell aplasia (25), neutropenia (25), acquired hemophilia A (25), thrombocytopenia (25), hemolytic-uremic syndrome (25), pancreatitis (26), asymptomatic raise in amylase and lipase (26), renal insufficiency with nephritis (27), arthritis (28), and myocarditis (Tajiri and Ieda).

Contributors to this research topic in Frontiers in Pharmacology and Frontiers in Oncology describe the importance of understanding this new class of drugs and their unique toxicities. Other areas covered include a description the current understanding of the basic mechanism of immune dysregulation in cancer patients undergoing immune checkpoint inhibitor treatment as well as potential predictive strategies for future clinical practice (Anderson and Rapoport). A second manuscript describes an unusual patient with persistent pruritus and lichenoid reaction secondary to anti-PD1 checkpoint inhibitor managed with narrowband ultraviolet B phototherapy (Donaldson et al.). A third manuscript explains the management of gastrointestinal toxicity with special reference to the immune homeostasis in the gastrointestinal tract (Dougan) and lastly a meta-analysis describing the relative risk and incidence of immune checkpoint inhibitor related pneumonitis in patients with advanced cancer (Ma et al.).

It must be emphasized that IrAEs are usually low-grade and controllable; however, the reporting of these irAEs is generally suboptimal (29). Oncologists should be aware that there is a wide range of additional distinctive toxicities and side effects that can be unpredictable and severe in nature. As these agents will, in the future, be administered with targeted therapies, vaccines, chemotherapy or radiation therapy it is possible that the incidence and severity of these toxocities may change. The different mechanisms of action of anti-CTLA-4 and anti-PD-1/anti-PD-L1 antibodies resulted in the development of clinical studies investigating combination therapies in a variety of malignancies including metastatic renal cell cancer and metastatic malignant melanoma. The incidence of serious grade 3 and grade 4 adverse events due to the combination of ipilimumab and nivolumab were present in approximately half of patients. The incidence of these toxicities was significantly higher than either antibody administered separately resulting in treatment interruption in one-third of patients (30). Clinical recommendations for managing irAEs arise from general clinical consensus and experience, as there are no prospective trials to assess whether one treatment strategy is superior to another. Although controversial; there are reports suggesting that the development of irAEs is associated with improvement in survival in patients with advanced or recurrent malignancy treated with immune checkpoint inhibitors (31).

Finally, early detection of IrAEs and proactive and adressive management by clinicians is critical to lower morbidity and mortality.

## Author Contributions

The author confirms being the sole contributor of this work and has approved it for publication.

### Conflict of Interest Statement

MDS: Advisory Board and Speaker Engagements; BMS: Advisory Board and Speaker Engagements; AstraZeneca: Advisory Board and Speaker Engagements.
